# Profit-Driven Adaptive Moving Targets Search with UAV Swarms

**DOI:** 10.3390/s19071545

**Published:** 2019-03-30

**Authors:** Xianfeng Li, Jie Chen, Fan Deng, Hui Li

**Affiliations:** 1 Shenzhen Key Lab of Information Theory & Future Network Arch, Peking University Shenzhen Graduate School, Shenzhen 518055, China; chen-jie@pku.edu.cn (J.C.); dengfan@pku.edu.cn (F.D.); lih64@pkusz.edu.cn (H.L.); 2 Future Network PKU Lab of National Major Research Infrastructure, Peking University Shenzhen Graduate School, Shenzhen 518055, China

**Keywords:** unmanned aerial vehicle (UAV), moving targets search, observation profit

## Abstract

This paper presents a novel distributed algorithm for a moving targets search with a team of cooperative unmanned aerial vehicles (UAVs). UAVs sense targets using on-board sensors and the information can be shared with teammates within a communication range. Based on local and shared information, the UAV swarm tries to maximize its average observation rate on targets. Unlike traditional approaches that treat the impact from different sources separately, our framework characterizes the impact of moving targets and collaborating UAVs on the moving decision for each UAV with a unified metric called *observation profit*. Based on this metric, we develop a profit-driven adaptive moving targets search algorithm for a swarm of UAVs. The simulation results validate the effectiveness of our framework in terms of both observation rate and its adaptiveness.

## 1. Introduction

Due to their high mobility and flexibility, unmanned aerial vehicle (UAV) systems with sensors onboard can be organized to carry out monitoring alike tasks without carrying any human personnel [1]. In the past decade, we have witnessed increasing military and civilian applications such as wind estimation, managing wildfire, disaster monitoring, remote sensing, traffic monitoring, border surveillance and mobile wireless coverage, etc. [2,3,4,5]. These applications often require a team of cooperating UAVs to continually track one or more moving entities [6,7,8]. For example, in the case of search and rescue task, the rescued agents should be kept under constant observation to ensure their safety. However, since the observation sensors of UAVs have limited sensing range and the number of UAVs might be less than that of moving targets, these UAVs should cooperate with each other in information sharing and path planning to maximize the number of targets under observation.

Along the main thread of the related research, the problem of moving targets search by UAV swarms has been characterized as a special form of Cooperative Multi-robot Observation of Multiple Moving Targets (CMOMMT) problem formalized in Ref. [9]. In the CMOMMT model, a team of homogeneous mobile robots is deployed to observe a set of targets moving within a restricted area of interest. The goal is to keep as many targets as possible under observation by at least one of the robots. In traditional CMOMMT approaches, the robots make decisions based on two separate factors: the targets currently under observation by a robot, and the need for coordination with other UAVs [9,10]. As far as we know, all the previous works consider these factors separately, and UAV operating decisions are not made from a clear metric.

The original contribution of this paper is a novel distributed algorithm called PAMTS, which stands for Profit-driven Adaptive Moving Targets Search with UAV Swarms. In PAMTS, the search region is first partitioned into a set of equally sized cells, and an observation history map for each cell is associated with individual UAVs. The history maps can be exchanged among UAVs within communication ranges to enrich their global knowledge, such that better moving decisions can be made. The decision making involves a trade-off between two intentions: the *follow* intention, which means keeping track of targets currently under observation; and the *explore* intention, which means searching recently unexplored cells for discovering more targets. The weights on the two intentions are implicitly captured by *profit-of-follow* (PoF) and *profit-of-explore* (PoE) respectively at each cell, and they will be combined into a single metric called *observation profit*. With this metric value for each cell calculated, the UAV will be able to make decisions on where to move by picking a destination cell with the best observation profit.

The rest of this paper is organized as follows. In Section 2, we discuss the related studies in multiple moving targets search. The problem formulation is introduced in Section 3. Section 4 describes our algorithm in detail. In Section 5, simulation results are presented. Finally, we conclude the paper in Section 6.

## 2. Related Studies

Moving targets search with a UAV swarm has attracted increasing research attention [11,12,13,14,15]. In essence, to effectively observe multiple moving targets, UAVs in the swarm should cooperate with each other like teammates [16,17,18].

**Figure 1:**
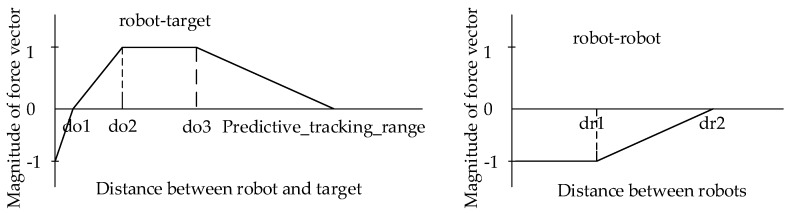
Magnitude of the force vectors from robot to target and robot to robot.

In a broader context, this can be viewed as a special form of the Cooperative Multi-robot Observation of Multiple Moving Targets (CMOMMT) problem, which is NP-hard [9]. In essence, robots work in search or track mode to maximize their observation coverage on targets. Mode switching is based on the presence of targets in the field of view (FOV) of each robot. On the one hand, when a robot finds one or more targets in its FOV, it changes to track mode and moves toward the center of mass of all the detected moving targets. On the other hand, when the robot is not monitoring any target, the robot switches to search mode for seeking a new target. The local force vectors, introduced in Refs. [9,10], suggest that a robot is attractive by nearby targets and repulsive by nearby robots. The calculation of the local force vectors is shown in Figure 1. To reduce overlapping observations on the same target, Paker [10] extends their initial work with a new approach called A-CMOMMT, which is based on the use of weighted local force vectors. In Refs. [19,20], the authors propose a behavioral solution with an algorithm, called B-CMOMMT, which adds the help mode of operation to reduce the risk of losing a target. A robot that is about to lose a target broadcasts a help request to other robots and the robots in search mode respond to this request by approaching the requester. Furthermore, personality CMOMMT (PCMOMMT) [21] uses the information entropy to balance the contradiction between the individual benefit and the collective benefit. More recently, C-CMOMMT [22] proposes an approach based on contribution in which each robot is endowed a contribution value derived from the number of assigned targets to it. Robots with low contribution receive strengthened repulsive forces from all others and robots with high contribution receive weakened attractive forces from low-weighted targets.

Besides using local force vectors, some other techniques have also been investigated. For example, model-predictive control strategies are used for CMOMMT in Ref. [23], but they have much higher computational complexity. The authors in Ref. [24] extend the conventional CMOMMT problem with fixed-altitude or fixed-FOV-size to multi-scale observations by using a multi-MAV system with noisy sensors. The authors in Ref. [25] replace the use of local force vectors with the introduction of a tracking algorithm based on unsupervised extended Kohonen maps. In Refs. [26,27], the authors present a novel optimization model for CMOMMT scenarios which features fairness of observation among different targets as an additional objective.The authors in Ref. [28] extend the conventional CMOMMT problem with limited sensing range and the moving targets are un-directional. In Ref. [29], the authors incorporate a multi-hop clustering and a dual-pheromone ant-colony model to optimize the target detection and tracking problem. The authors in Ref. [30] utilize the Mixed Integer Linear Programming (MILP) techniques to arrange the UAVs to perform city-scale video monitoring of a set of Points of Interest (PoI).

Since the above algorithms consider the impact of moving targets and collaborating UAVs separately, they fail to provide an elegant framework for making trade-offs among target searching and target tracking for each UAV. In our earlier conference paper [31], we presented our initial efforts to make trade-offs between target *searching* and *tracking* in a single framework. In this work, we extend this framework with more comprehensive investigations and experiments. By characterizing each cell with a changing observation profit, both of the impact factors of moving targets and collaborating UAVs are considered in a unified framework. With this framework, a profit-driven algorithm that makes moving decisions for each UAV can be designed conveniently by picking observation cells with the best observation profit.

## 3. Problem Formulation

**Figure 2:**
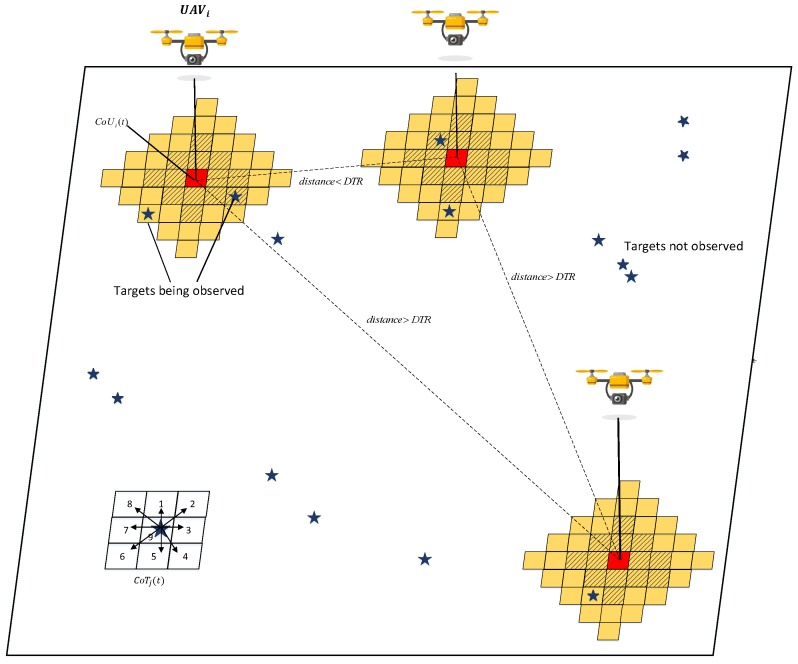
UAVs and targets (stars) are moving in a bounded area that is partitioned into cells; $CoU_{i}\left(t\right)$ and $CoT_{j}\left(t\right)$ represent the locations of UAV *i* and target *j* at time *t*, respectively; the yellow shaded areas denote the FOV (field of view) of each UAV when SR=4 while the slash shaded areas represent the movement choices for each UAV at the next time step; UAVs can communicate with each other within data transmission range DTR.

Figure 2 illustrates the problem of Cooperative Multi-UAV Observation of Multiple Moving Targets, with some concepts and terms introduced as follows.

**Search Region**. The search region is considered to be a 2D bounded area partitioned into *C* equally sized cells, and each UAV is aware of its cell location with its onboard GPS sensors.

**Time Steps**. We define symbol *t* as the discrete time step within which UAVs can make a move-and-observe action.

**Targets**. The mission is about observing a set of *M* moving targets in the search region. For target *j*, the term $CoT_{j}\left(t\right)$ is the **C**ell **o**f **T**arget *j* at time step *t*. We assume and the velocity of targets is smaller than that of UAVs. More specifically, each target can move into one of its eight neighbor cells or stay at its current cell at time step t+1.

**UAVs**. A set of *N* UAVs are deployed in the search region to observe the *M* targets (we assume N<M). The term $CoU_{i}\left(t\right)$ denotes the **C**ell **o**f **U**AV *i* at time step *t*. With the help of onboard sensors, UAVs can act as a team to share information and coordinate with each other for more efficient target searching. Each UAV is equipped with several other sensors as follows:

- A GPS sensor to provide geographical location for the UAV;
- An omnidirectional sensor for target observation. Let SR be the sensing range of each UAV, and its field of view (FOV) of UAV *i* at time step *t* is represented as a subset of contingent cells $FOV_{i}\left(t\right)$ centered in $CoU_{i}\left(t\right)$.
- A wireless network interface for communication with other UAVs. Assume the data transmission range is DTR, which is larger than the sensing range SR of the UAVs.
- A computing unit onboard for execution of the search algorithm.

**Observation History Map**. Each UAV maintains an observation history map which records two pieces of information: (1) the last observed time for each cell *c*, denoted as a timestamp $stamp_{i}^{c}\left(t\right)$, and (2) the locations of observed targets, if any. At each time step, UAV *i* makes observation on the subset of cells $FOV_{i}\left(t\right)$, then the values of corresponding cells in the observation history map will be updated with the latest observation. In addition to updates by local observation, the exchange of observation history maps among UAVs will also trigger updates on the local map of each UAV. One of the benefits is that a UAV *i* can avoid wasting its effort on observing a subarea which has just been observed by other UAVs without discovering any target.

**Objective**. A target *j* can be monitored by more than one UAV at time step *t*, and we define the observation state of target *j* as $O_{j}\left(t\right)$:

(1) $$\begin{array}{c} O_{j}\left(t\right)=\left\{\begin{array}{c} 1,\;\;\;\;\;if\;\exists UAVi:CoT_{j}\left(t\right)\in FOV_{i}\left(t\right),\\ 0,\;\;\;\;\;otherwise, \end{array}\right. \end{array}$$

where $O_{j}\left(t\right)=1$ means that target *j* is observed by at least one UAV at time *t*, and $O_{j}^{t}=0$, otherwise.

The objective of this work is to develop an algorithm to maximize the average observation rate of *M* targets in a period of *T* time steps, which can be characterized by the metric Θ:

(2) $$\Theta \left(T\right)=\frac{1}{M*T}\sum_{t=1}^{T}\sum_{j=1}^{M}O_{j}\left(t\right).\;$$

Ideally, Θ can reach its maximum value 1 when $O_{j}\left(t\right)$ is 1 for every target *j* at every moment *t*. However, as given by Formula (1), $O_{j}\left(t\right)$ is constrained by the locations of targets as well as the field of view of UAVs; therefore, it is unrealistic for $O_{j}\left(t\right)=1$ for every target *j* at every time *t*. In other words, Formula (1) serves as constraints for the objective function in Formula (2), and our task is to maximize the number of $O_{j}\left(t\right)=1$ situations.

For ease of comprehension, the notations introduced above, along with some more notations in subsequent sections, have been put in Table A1 in the Appendix of this paper.

## 4. Algorithm

**Figure 3:**
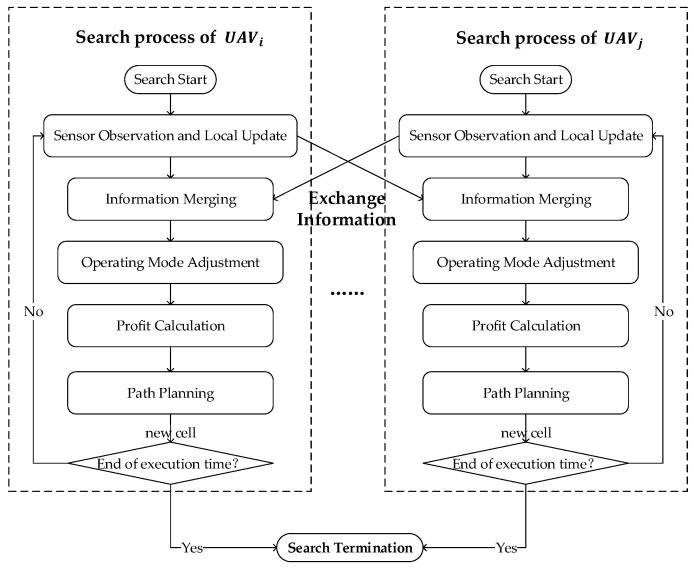
The framework of PAMTS.

We formulate the CMOMMT algorithm as follows. First, the operation of each UAV can be characterized as a trade-off between two intentions: the *follow* intention, which keeps tracking of targets currently under observation; and the *explore* intention, which searches recently unexplored cells to discover more targets. Depending on the recent observation history, a UAV can adjust its weights of intentions, which will be characterized by the unified *observation profits* on the cells. Intuitively, a cell with higher profit means the area surrounding it deserves observation more than a cell with a lower profit in terms of the *follow*/*explore* intensions, and the detailed definition and computation of the *observation profits* will be given in Section 4.4. Driven by this metric, UAVs will make decisions by moving towards cells with higher profits. The high-level framework is shown in Figure 3, which mainly consists of five components: (i) Sensor observation and local update; (ii) Information merging; (iii) Operating mode adjustment; (iv) Profit calculation; and (v) Path planning. We elaborate on each of these components as follows.

### 4.1. Sensor Observation and Local Update

At each time step *t*, each UAV will make observations on the targets within its FOV and update its observation history map, including the observing times of cells and the positions of the observed targets. With the completion of local update, each UAV should broadcast its observation history map to other UAVs and meanwhile receive broadcasts from teammates to trigger the information merging process.

### 4.2. Information Merging

We assume that the information exchange is restricted among UAVs within their data transmission range. Therefore, the observation history maps may not be consistent among all UAVs. Based on the distance among UAVs, the whole UAV swarm will be divided into serval sub teams and the UAVs in the same team will share the information. We enforce the following rules for information merging. First, when a target *j* is observed by more than one UAV at time *t*, we tag its tracker to be the closest UAV, denoted as $tracker_{j}\left(t\right)$. This tracker UAV will take the responsibility for following the target in the next time step, and this responsibility will be reflected in its observation profit calculation. Other UAVs in the team that also observed this target will then delete the position information of target *j* to guarantee that they will not be affected by this target.

Second, when the timestamp of a cell received from broadcast is newer than that in the local observation history, it will be updated accordingly. Therefore, after information merging, the observation history map of each UAV contains two pieces of information: (1) observation timestamps of cells, (2) the targets that should be tracked in the next time step and their positions.

Overall, in addition to the observation history map, an *observation summary* for each UAV *i* derived from information merging is also maintained, which includes the following items:

- The number of targets that UAV *i* should track at time *t*, denoted as $NoT_{i}\left(t\right)$;
- The number of UAVs in the sub team which contains UAV *i* at time *t*, denoted as $NoU_{i}\left(t\right)$;
- The total number of targets observed by UAV *i* and its teammates that have exchanged information with it at time *t*, denoted as $\pi_{i}\left(t\right)$. Note that this number is not equal to the total number of observed targets by the whole UAV swarm because some observations may not get exchanged among UAVs beyond their communication ranges.

### 4.3. Operating Mode Adjustment

In conventional CMOMMT search strategies, when a UAV finds one or more targets in its FOV, it moves toward the virtual center of the observed targets to *follow* them; otherwise, it will perform an *explore* action on unobserved cells to try to find targets. However, when fewer UAVs are deployed to observe a larger number of targets, each UAV may need to take both the responsibility of *follow* action and *explore* action in case the number of targets observed by it is less than a threshold, e.g., M/N. Otherwise, if each UAV is just following a single target in dedication, there will be no UAV spending its effort to discover the rest unobserved targets (M−N in the worst case).

Instead of switching between the *follow* and *explore* actions, our framework enables a more flexible adjustment of operating modes. It takes *follow* and *explore* as intentions rather than actions, and a trade-off on the degrees of the two intentions is made based on the observation histories of UAVs. Intuitively, a UAV currently with more targets under observation will have a higher *follow* intention, which will make the UAV to move towards cells containing the observed targets. Otherwise, if a UAV has very few targets under observation, it will have a higher *explore* intention, and will move towards a direction with more unobserved cells by itself as well as its teammates recently.

To make quantitative trade-off, we introduce two parameters—α and β—to characterize the intentions for *follow* and *explore*, respectively. The calculation of the two parameters is actually decided by two levels of information: the local number of observed targets by a UAV, and the global number of observed targets by all UAVs. We take the global level into account because each UAV will have a higher responsibility to *explore* unobserved cells if only a small number of targets are observed by the whole UAV swarm. This means that, for the same number of targets being observed by a UAV, it may spend different effort for *explore*, depending on the total number of observed targets by the whole team.

Table 1 gives the details on how the local parameters are decided for each UAV at a specific time *t*. Since the overall search system is discretized in both time and space, the parameter values are in discretized levels as well. In particular, we divide the number of targets observed by a UAV into five levels. If the number of targets observed by UAV *i* is above M/N (which is the average number of targets that should be observed by each UAV), we will have $\alpha_{i}^{local}\left(t\right)=1$ and $\beta_{i}^{local}\left(t\right)=0$. This means that the effort of UAV *i* will be entirely on following the observed targets, and it corresponds to level 1 in Table 1. In essence, the less number of targets observed a UAV, the less value of α and the more value of β are assigned to that UAV.

At the global level, we use the merged information to make adjustments on the efforts that should be spent on *follow* and *explore*, respectively. In particular, we use $\pi_{i}\left(t\right)$ to adjust its search efforts. To reflect the quality of its knowledge on observed targets, $NoU_{i}\left(t\right)$ is also included to calibrate the calculation. The concrete formulas for global level parameters are given as follows:

(3) $$\alpha_{i}^{global}\left(t\right)=\frac{\pi_{i}\left(t\right)}{NoU_{i}\left(t\right)\times M/N},\;$$

(4) $$\beta_{i}^{global}\left(t\right)=1-\frac{\pi_{i}\left(t\right)}{NoU_{i}\left(t\right)\times M/N}.$$

By combining the parameters at both the local and global levels, the final parameters for follow and explore are calculated as follows:

(5) $$\alpha_{i}\left(t\right)=\alpha_{i}^{local}\left(t\right)\times \alpha_{i}^{global}\left(t\right),\;$$

(6) $$\beta_{i}\left(t\right)=\beta_{i}^{local}\left(t\right)\times \beta_{i}^{global}\left(t\right).\;$$

### 4.4. Profit Calculation

As described earlier, unlike existing techniques that consider UAV-target and UAV-UAV relationships separately, our work provides a single framework to account for the impact from the two aspects. For this purpose, we introduce a key metric called *observation profit* that unifies the parameters α and β, and this metric is not applied to individual UAVs or targets. Instead, *observation profit* is used to characterize the cells for observation, and UAVs will move towards nearby cells with higher observation profits. The *observation profit* is calculated from two aspects for each cell: *profit of follow* (PoF) and *profit of explore* (PoE).

**Figure 4:**
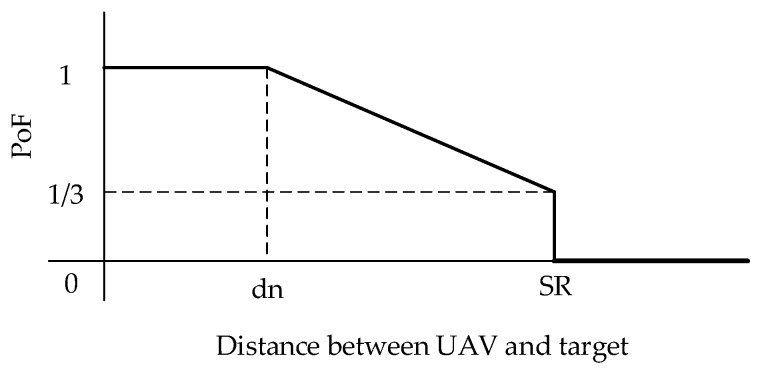
The calculation on PoF (profit of follow) contribution by a target.

**Calculation of PoF.** For the CMOMMT problem, due to the high mobility of targets and UAVs, an observed target may escape from the UAV’s FOV at the next time step. Thus, targets in different locations will contribute different PoF values to the observing UAV. Intuitively, a target in the middle of the UAV’s FOV contributes higher profit of follow than another target in the edge area, as the former target has lower chances to escape observation. Figure 4 presents the concrete method for calculating PoF contribution according to the distance between the UAV and a target. The distance dn is a threshold in which targets will have no chance to escape observation in the next time step even in the worst case (where the UAV and the target are moving apart at their maximum speed). Targets with a distance between dn and SR from the UAV will have increasing chances to escape observation in the next time step, and targets beyond this distance will have no chance to be observed, thus they will have no contributions to PoF.

Let $\sigma_{i}\left(t\right)$ be the subset of cells to which UAV *i* can move in one time step, which is a surrounding area of its current cell $CoU_{i}\left(t\right)$. For each potential destination $c\in \sigma_{i}\left(t\right)$, its PoF value will be the summation of PoF contributions from targets within distance SR. A higher PoF value for cell *c* means that UAV *i* can observe more targets if it moves to *c* at time t+1. The detailed PoF calculation for cells is described together with the calculation of another metric PoE in Algorithm 1.

**Algorithm 1:**PoF and PoE Calculation **Input:** (1) Parameters of the search mission (*M*, *N*, etc); (2) Observation history map for each UAV (depicted in Section 3); (3) Observation summary for each UAV (depicted in Section 4.2) 
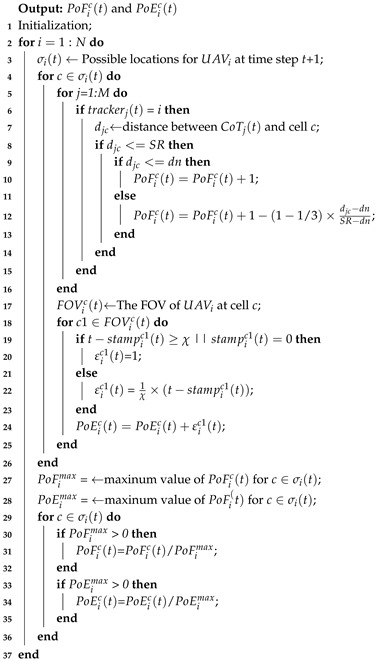


**Calculation of PoE.** In addition to tracking observed targets, another task for UAVs is to discover targets currently not being observed. Intuitively, a cell that has never been observed should have the highest value 1 for exploration, and a cell that has just been observed should not be observed immediately, thus having value 0 for exploration. With time elapsed, the chance a target moving to the observed cell gets increased gradually, and its exploration value should be recovered to 1 gradually as well. Since a UAV *i* knows the last observation time $stamp_{i}^{c}\left(t\right)$ of cell *c* from its observation history map, and this knowledge might be outdated, we introduce a term $\varepsilon_{i}^{c}\left(t\right)$ to denote the exploration value for cell *c* at time *t* in the view of UAV *i*, which is calcuated as follows:

(7) $$\begin{array}{c} \varepsilon_{i}^{c}\left(t\right)=\left\{\begin{array}{c} 1,\;\;\;\;\;\;\;\;\;\;\;\;\;\;\;\;\;\;\;\;\;\;\;\;\;\;\;\;\;\;\;\;\;\;\;\;\;\;\;\;\;\;\;\;\;\;\;t-stamp_{i}^{c}\left(t\right)\geq \chi \;or\;initial\;value,\\ \frac{1}{\chi }\times \left(t-stamp_{i}^{c}\left(t\right)\right),\;\;\;\;\;otherwise, \end{array}\right. \end{array}$$

where χ decides the window of time steps that the exploration value of a cell is recovered from 0 to 1. This is to reflect the fact that the longer the elapsed time since the last observation on some cell, the more chances a target moved to this cell, thus the higher exploration value for this cell.

With the exploration values for individual cells defined above, we can calculate the *profit of explore* (PoE) for a destination cell *c* for UAV *i* at time t+1 by summing up the exploration values of cells around *c*. The higher PoE for *c*, the more cells around it that deserve exploration by the UAV. Note that these surrounding cells actually correspond to the FOV of UAV *i*, assuming it is flying above *c*. Algorithm 1 gives the details on PoE calculation together with PoF discussed earlier.

**Calculation of*****observation profit*****.** With PoF and PoE calculated, we proposed an objective function to calculate the *observation profit* of each cell $c\in \sigma_{i}\left(t\right)$ for $UAV_{i}$ as follows:

(8) $$\rho_{i}^{c}\left(t\right)=\alpha_{i}\left(t\right)\times PoF_{i}^{c}\left(t\right)+\beta_{i}\left(t\right)\times PoE_{i}^{c}\left(t\right).$$

In this formula, the trade-off between *follow* and *explore* is calibrated by the two parameters α and β calculated the operating mode adjustment step.

### 4.5. Path Planning

Path planning is about deciding a destination cell $c\in \sigma_{i}\left(t\right)$ for UAV *i* at time *t*, to which UAV *i* will fly at time t+1 to make observations. Since we have calculated the *observation profit* for each cell in $\sigma_{i}\left(t\right)$, we can easily make decisions by choosing the cell with the maximum *observation profit*.

### 4.6. Complexity Analysis

For a given UAV *i* in cell *c* at time step *t*, assuming that the maximum velocity of UAV is *v*, it is easy to determine that the maximum number of cells in subset $\sigma_{i}\left(t\right)$ can be represented as mathematical expression: $4v^{2}-4v+5$. Similarly, the maximum number of cells in subset $FOV_{i}\left(t\right)$ is $4{SR}^{2}-4SR+5$. According the the proposed algorithm, the computational complexity at each UAV is listed in Table 2.

As for the space complexity, each UAV maintains an observation map and an observation summary. As mentioned in Section 4.2, the observation summary only needs to store three numbers, thus it consumes negligible memory. As described in Section 3, the observation history map for each UAV contains two sets of information, the observation timestamp consumes O(C) memory, and the locations of observed targets consume O(M) memory. Therefore, the total amount of memory consumption at each UAV is in the level of O(C)+O(M).

## 5. Experiment Results

We perform simulations to validate the effectiveness of the proposed algorithm, and compare its performance with other approaches, including A-CMOMMT [10], B-CMOMMT [19], C-CMOMMT [22] and random walk. Note that the first three works use the traditional CMOMMT architecture which are based on local force vectors, and the random walk chooses UAV location at each step by randomly selecting one of its adjacent cells. Random walk does not make use of any history information or current status except for its current location, and always serves as the performance baseline of the target search missions.

### 5.1. Experimental Setup

Simulations have been carried out in Matlab (R2017a(9.2.0.538062), The Mathworks, inc. Natick, MA, USA). For ease of comparison, the parameters of simulations are carefully set to be similar with the values in Refs. [10,19,22]. According to the application scenario described in Figure 2, we use the following parameters for concrete simulation: the size of the search region is set to 40,000 × 40,000 units, and the size of each cell is 100×100 units. Therefore, the search region is actually composed of 400×400 cells. At the start of each simulation run, the *N* UAVs and *M* targets are randomly distributed in the search area. The maximum UAV speed is set to 200 units (2 cells) per time step, and 100 units (1 cell) for target speed. UAV sensor parameters include sensing range of SR=2600 units (26 cells) and data transmission range of DTR=5000 units. The recovery window of exploration value is set as χ=5, which means a cell observed after five time steps will recover to observation value of 1.

### 5.2. Comparison with Other Approaches

**Figure 5:**
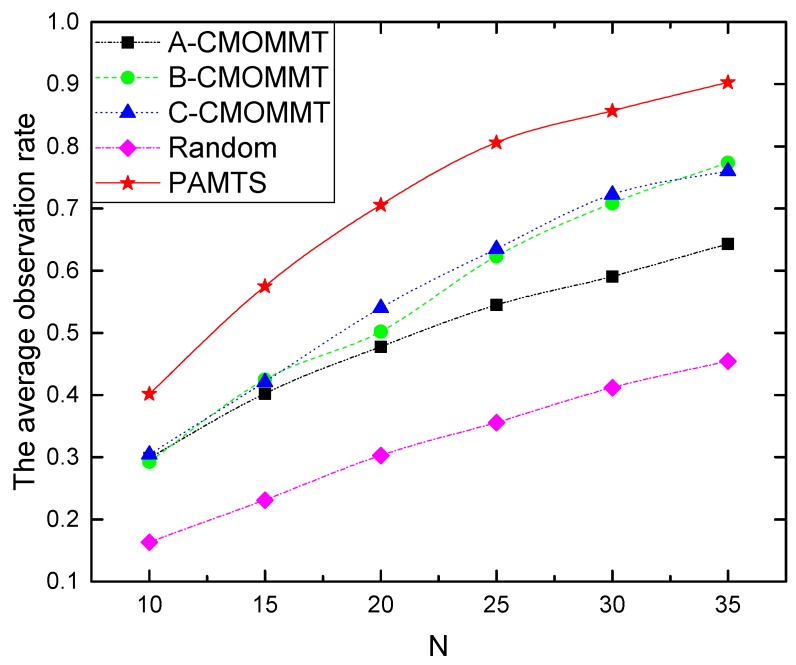
The impact of the various number of UAVs deployed in the search mission while that of targets is fixed to 40.

We first compare our work against related works with different numbers of UAVs deployed to search 40 targets. As shown in Figure 5, the average observation rate improves with the increased number of UAVs across all approaches. In addition, our work (PAMTS) is consistently better than any other methods. The concrete improvements of PAMTS over the other methods are presented in Table 3. It can be seen that the improvement is above 30% in most cases.

**Figure 6:**
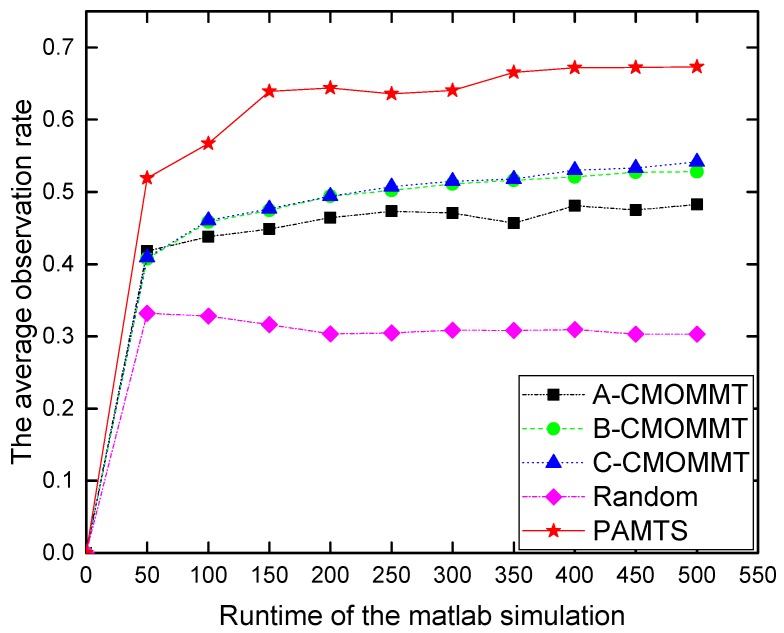
The saturation of the performance with increasing runtime under the condition that N/M= 20/40.

We then study the variations of observation rate with increasing runtime. In this experiment, we set the UAV/target ratio at 20/40. Figure 6 shows that A-CMOMMT, B-CMOMMT, C-CMOMMT and random walk stops improvement fairly early, while our PAMTS algorithm continues to improve performances for a longer time. This suggests that efficient information sharing in PAMTS enables more potentials for optimized deployment of UAVs in the search region.

**Figure 7:**
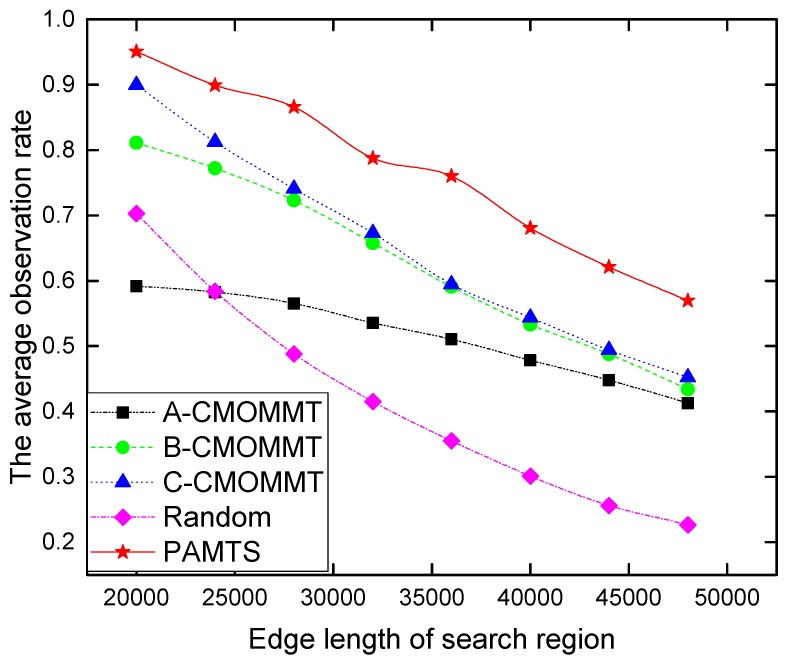
The impact of various size of search region on search performance under the condition of N/M= 20/40 and SR= 2600 units.

We also study the impact of the size of the search region on search performance under the condition that the ratio of the UAV/target ration is fixed at 20/40 and the sensing range of UAVs also keeps unchanged at SR=2600 units. It can be seen in Figure 7 that the observation rate goes down with larger search areas because targets are more scattered and it becomes more difficult for UAVs to keep track of targets. Nevertheless, PAMTS still exhibits its advantages over the other methods under more challenging environments.

### 5.3. Adaptability Analysis

Having proved the advantages of our work over the related work under different situations, we further investigate the impact of a couple of parameters on our algorithm. In particular, since our work relies on information exchange among UAVS for better target observations, we would like to study how significant the effect of communication range is on search performance. We set N/M ratio at 20/40, and sense range SR=2600.

**Figure 8:**
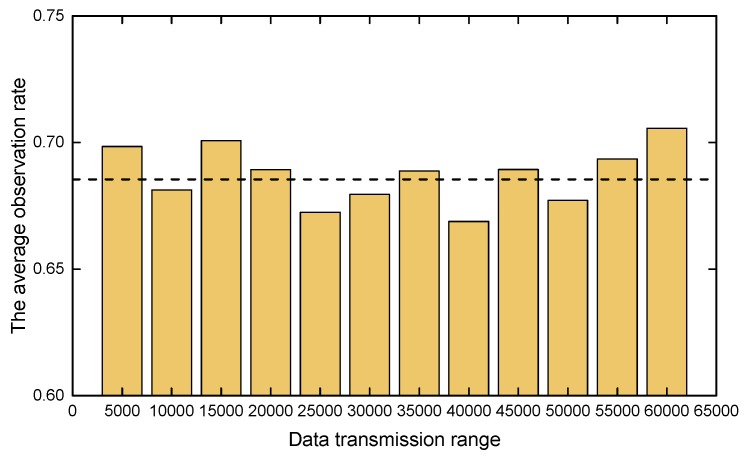
The impact of different communication conditions on search performance under the condition of N/M= 20/40 and SR= 2600 units.

Figure 8 shows that the search performance does not improve with increasing data transmission range. This appears to be non-intuitive, as longer communication range means more sufficient exchange of information among UAVs. However, since UAVs as mechanical gadgets can only move in a relatively small range for each time step, only information from nearby UAVs is useful for making decisions by UAVs. Thus, exchanging information with remote teammates does not provide appreciable benefits. Sometimes, the aggregated impact from a remote area which the UAV cannot reach in the near future can even mislead the UAV for making decisions in some degree. Therefore, a communication infrastructures with moderate wireless link range is enough for our work.

**Figure 9:**
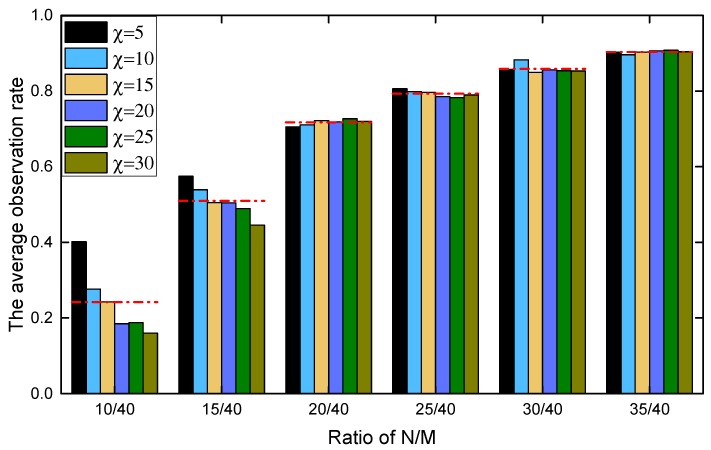
The effect of parameter χ on search performance.

Another parameter that may have an impact on performance is recovery window of exploration value χ. Its default value is set to 5, which means an observed cell will recover to its full exploration value after five time steps. Figure 9 reports the impact of χ under different N/M settings. One conclusion is that slower recovery is not beneficial—the reason is that targets may move into a cell observed previously, and changes the observation value for that cell. Therefore, slow recovery does not respect this dynamics and may result in worse performance. Another conclusion is that, with higher N/M ratio, the setting on the recovery window has decreasing impact, as denser distribution of UAVs will naturally improve the chances for exploring observed cells before their observation value recovered to a high one. Overall, this experiment shows that setting χ at 5 is a good choice.

## 6. Conclusions

In this paper, we designed a novel algorithm for moving targets search with cooperative UAVs. Unlike traditional CMOMMT approaches that handle UAV-target and UAV-UAV relationships separately, our framework considers the two relationships in a unified framework. This is achieved by introducing a key concept called *observation profit* of cells. Based on this unified metric, we designed a Profit-driven Adaptive Moving Targets Search framework, called PAMTS. In this framework, a trade-off between the two UAV operating actions, follow and explore, can be made conveniently. The simulation results show that the search performance of PAMTS is significantly better than traditional CMOMMT approaches. In addition, by adjusting a set of parameters, PAMTS can be adaptable to various searching scenarios conveniently.

## Figures and Tables

**Table 1:** The relationship between $Num_{i}\left(t\right)$ and local coefficients.

Level	Description	$\alpha_{i}^{\mathrm{local}}\left(t\right)$	$\beta_{i}^{\mathrm{local}}\left(t\right)$
1	$NoT_{i}\left(t\right)>=M/N$	1	0
2	$3M/4N<=NoT_{i}\left(t\right)<M/N$	3/4	1/4
3	$M/2N<=NoT_{i}\left(t\right)<3M/4N$	1/2	1/2
4	$M/4N<=NoT_{i}\left(t\right)<M/2N$	1/4	3/4
5	$NoT_{i}\left(t\right)<M/4N$	0	1

**Table 2:** Complexity analysis of each parts of the proposed algorithm.

Part	Algorithm Time Complexity
Sensor Observation and Local Update	O(M)+O(C)
Information Merging	O(NC)
Operating Mode Adjustment	O(1)
Profit Calculation	$O(Mv^{2})$+$O({\left(SR\right)}^{2}v^{2})$
Path planning	$O({\left(SR\right)}^{2})$

**Table 3:** The improvement on observation rate of our proposed algorithm over A-CMOMMT, B-CMOMMT, C-CMOMMT and random walk for M= 40, N= 10, 15, 20, 25, 30, 35.

M	N	A-CMOMMT	B-CMOMMT	C-CMOMMT	Random Walk
40	10	34.38%	37.14%	31.96%	145.92%
	15	42.91%	35.42%	36.46%	148.71%
	20	47.77%	40.62%	30.60%	133.2%
	25	48.02%	29.32%	26.92%	126.56%
	30	45.20%	21.01%	18.66%	108.15%
	35	40.43%	16.73%	18.77%	98.49%
Average Values		43.12%	30.04%	27.23%	126.85%

## Abbreviations

The following abbreviations are used in this manuscript:

UAV	Unmanned Aerial Vehicle
CMOMMT	Cooperative Multi-robot Observation of Multiple Moving Targets
PAMTS	Profit-driven Adaptive Moving Targets Search with UAV Swarms
FOV	Field of View
PoF	Profit of Follow
PoE	Profit of Explore

## Appendix A

This appendix is the list of notations used in paper.

**Table A1:** Notations used in paper.

Notation	Description	Notation	Description
*M*	number of moving targets	*N*	number of UAVs
*t*	time step	$CoT_{j}\left(t\right)$	cell in which target *j* is located at time step *t*
SR	sensing range of each UAV	$CoU_{i}\left(t\right)$	cell in which UAV *i* is located at time step *t*
DOT	data transform distance of each UAV	$FOV_{i}\left(t\right)$	field of view of UAV *i* at time step *t*
Θ	average observation rate	$O_{j}\left(t\right)$	observation state of target *j* at time step *t*
$tracker_{j}\left(t\right)$	UAV which will track target *j*	$NoU_{i}\left(t\right)$	number of UAVs in the sub team of UAV *i*
$stamp_{i}^{c}\left(t\right)$	last observed time of cell *c*	$\pi_{i}\left(t\right)$	number of targets observed by the sub team of UAV *i*
υ	maximum velocity of UAV	$NoT_{i}\left(t\right)$	number of targets tracked by UAV *i*
α	parameter of follow intention	β	parameter of explore intention
PoF	profit of follow intention	$\varepsilon_{i}^{c}\left(t\right)$	exploration value of cell *c* at time step *t*
PoE	profit of explore intention	χ	recovery time window of exploration value
$\rho_{i}^{c}\left(t\right)$	observation profit of cell *c* at time step *t*	$\sigma_{i}\left(t\right)$	subset cells that UAV *i* can move in one time step
